# Spontaneous pneumomediastinum, pneumopericardium, pneumothorax and subcutaneous emphysema in patients with COVID-19 pneumonia, a case report

**DOI:** 10.1186/s13019-020-01308-7

**Published:** 2020-10-07

**Authors:** Vikisha Hazariwala, Hind Hadid, Denise Kirsch, Cecilia Big

**Affiliations:** Beaumont Hospital, Dearborn, MI USA

**Keywords:** COVID-19 pneumonia, Spontaneous pneumomediastinum, Spontaneous pneumopericardium, Acute respiratory distress syndrome, Severe acute respiratory syndrome

## Abstract

**Background:**

Spontaneous pneumomediastinum unrelated to mechanical ventilation is a newly described complication of COVID-19 pneumonia. The objective of this case presentation is to highlight an important complication and to explore potential predisposing risk factors and possible underlying pathophysiology of this phenomenon.

**Case presentation:**

We present two patients with COVID-19 pneumonia complicated by spontaneous pneumomediastinum, pneumopericardium, pneumothorax and subcutaneous emphysema without positive pressure ventilation. Both patients had multiple comorbidities, received a combination of antibiotics, steroids and supportive oxygen therapy, and underwent routine laboratory workup. Both patients then developed spontaneous pneumomediastinum and ultimately required intubation and mechanical ventilation, which proved to be challenging to manage.

**Conclusions:**

Spontaneous pneumomediastinum is a serious complication of COVID-19 pneumonia, of which clinicians should be aware. Further studies are needed to determine risk factors and laboratory data predictive of development of spontaneous pneumomediastinum in COVID-19 pneumonia.

## Background

As the 2019 coronavirus pandemic unfolded globally, numerous reports of severe and unusual complications in infected patients have been published. Acute respiratory distress syndrome (ARDS) is a major and sometimes fatal complication observed in as many as 41% of hospitalized patients with COVID-19 [1]. Acute renal failure, cardiomyopathy, and thromboembolic phenomena were also observed in COVID-19 patients in varying severities and percentages [2, 3].

The following cases outline the clinical course of two patients with COVID-19 pneumonia who developed spontaneous pneumomediastinum (SPM), pneumopericardium (SPP), pneumothorax, and subcutaneous emphysema without positive pressure ventilation. These findings are extremely rare with only a few similar cases in English literature to date [4, 5].

## Case presentation

### Patient 1

**Fig. 1:**
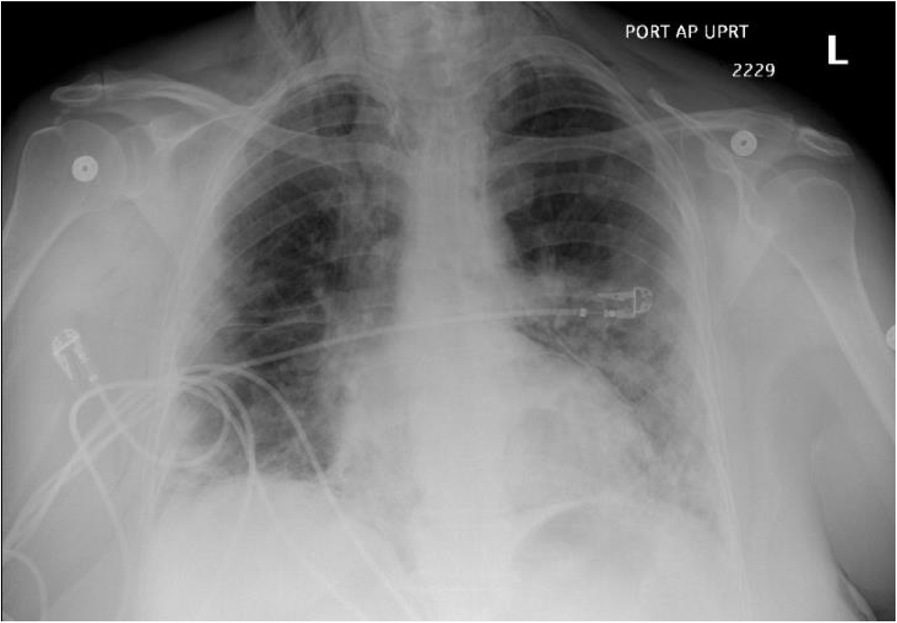
A frontal chest radiograph of Patient 1 showing interval development of pneumomediastinum, small pneumothoraces and subcutaneous emphysema

**Fig. 2:**
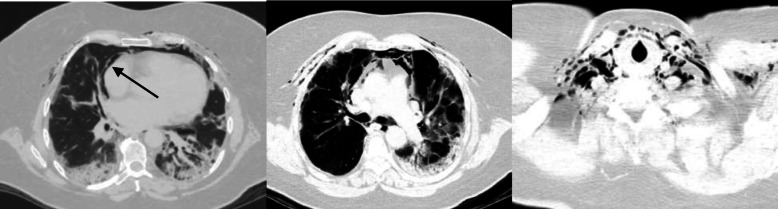
Computed tomography of the chest without contrast of Patient 1. There is a pneumopericardium (left) and extensive pneumomediastinum (middle) extending along soft tissues of the anterior and lateral chest wall as well as anterior neck soft tissues (right)

A 57-year-old Hispanic woman with asthma, hypertension and obesity (BMI 36 kg/m^2^) presented to the emergency department with 17 days of severe cough and progressive shortness of breath. She tested positive for COVID-19 via RT-PCR. She was saturating 93% on 4 L/min nasal cannula (NC). Her chest X-ray (CXR) showed bibasilar interstitial airspace disease. Initial laboratory workup revealed the following: WBC 5.7 thous/uL, platelets 212 thous/uL, procalcitonin 0.12 ng/mL, C-reactive protein 94 mg/L, fibrinogen 706 mg/dL, d dimer 541 ng/dL FEU, lactate dehydrogenase 459 U/L, and ferritin 1204 ng/mL. A five-day course of hydroxychloroquine (HCQ), zinc sulfate, and azithromycin was initiated, along with intravenous methylprednisolone 40 mg every 12 h and enoxaparin thromboprophylaxis. She continued to require increasing levels of supplemental oxygen and was placed on 15 L/min high flow nasal cannula (HFNC). A CXR performed on day ten showed pneumomediastinum, small bilateral apical pneumathorax, and subcutaneous emphysema (Fig. 1). A follow-up chest computed tomography (CT) revealed pneumopericardium and progression of multifocal interstitial opacities (Fig. 2). Her respiratory status continued to worsen and was intubated on day 16. Significant worsening of the subcutaneous emphysema was noted post-intubation. Cardiothoracic surgery was consulted and no acute intervention was recommended, as the patient had stable blood pressure and the pneumomediastinum was not believed to be a significant contributor to her respiratory deterioration. he continued to require increasing levels of oxygen and was subsequently flown to a tertiary care center for higher level of care. There, she was placed on extracorporeal membrane oxygenation (ECMO) and subsequently required bilateral chest tubes for enlarging pneumothoraces. Over the course of the next month, she could not wean off of ECMO and developed septic shock with profound lactic acidemia. Care was withdrawn per the request of her family and the patient expired immediately.

### Patient 2

**Fig. 3:**
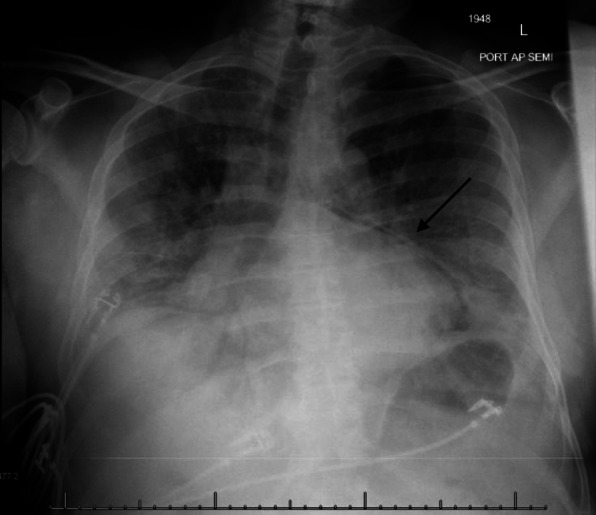
A frontal chest radiograph of Patient 2 showing interval development of spontaneous pneumomediastinum and possible pneumopericardium (arrow)

**Fig. 4:**
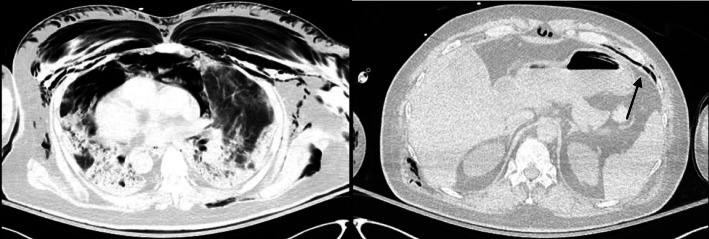
Computed tomography of the chest without contrast of Patient 2. A large pneumomediastinum and extensive soft tissue emphysema involving the anterior and posterior thorax (left). A tiny pneumoperitoneum in upper anterior abdomen (right)

A 55-year-old African American man with asthma, type II diabetes mellitus, hypertension, hyperlipidemia, obesity (BMI 30 kg/m^2^) and 50-pack-year smoking history presented to the emergency department complaining of cough, fevers and progressive dyspnea for 7 days. He tested positive for COVID-19 via RT-PCR. He was febrile at 101.0 °F with oxygen saturation of 84% on room air. He was placed on 5 L/min NC and admitted to the hospital. Initial CXR revealed patchy left basilar and right suprahilar airspace opacities. His laboratory workup was as follows: WBC 2.7 thous/uL, platelets 92 thous/uL, procalcitonin 8.15 ng/mL, C-reactive protein 116 mg/L, fibrinogen 440 mg/dL, d dimer 1917 ng/dL FEU, lactate dehydrogenase 503 U/L, and ferritin > 2000 ng/mL. He was initiated on a five-day course of HCQ, zinc sulfate, and azithromycin, along with intravenous methylprednisolone 40 mg every 12 h. Four days later, he continued to be febrile and required 15 L/min HFNC to maintain oxygen saturation ≥ 90%. On day nine, the patient developed pleuritic chest pain and an episode of non-sustained ventricular tachyarrhythmia. A repeat CXR showed interval development of pneumomediastinum (Fig. 3). On day ten, he was intubated due to progressive ARDS. Chest CT revealed extensive subcutaneous emphysema, large pneumomediastinum and adjacent small pneumoperitoneum (Fig. 4). Cardiothoracic surgery opted for conservative management as the patient was otherwise hemodynamically stable. On serial imaging, the subcutaneous emphysema worsened and his course was complicated by septic shock, acute systolic heart failure, stroke, ventilator-associated pneumonia, fungemia and an inability to wean off the ventilator. He suffered from cardiac arrest and passed away on day 50 of admission.

## Discussion and conclusions

Secondary pneumomediastinum and pneumopericardium are most often associated with chest trauma, esophageal perforation, cardiothoracic surgery or mechanical ventilation. SPM and SPP, however, are less common phenomena [6]. During the severe acute respiratory syndrome (SARS) outbreak in 2002–2003, a study from Hong Kong reported an incidence of 11.6% of SPM in infected patients [7]. The incidence of SPM and SPP in patients with COVID-19 pneumonia is unknown.

The pathophysiology of SPM in SARS patients is thought to be related to diffuse alveolar damage (DAD) which leads to gas leak into the pulmonary interstitium causing pneumomediastinum [7]. This concept is supported by postmortem studies on SARS lungs revealing extensive features of acute exudative alveolar and vascular injury [8]. Like the SARS virus, the novel COVID-19 virus causes ARDS in a sizable percentage of infected patients, but the mechanisms of alveolar injury are still under investigation. However, emerging autopsy reports of COVID-19 patients from Italy and the United States revealed that DAD appears to also be the predominate pathologic pattern of pulmonary injury in COVID-19 patients [9, 10].

Pulmonary barotrauma from mechanical ventilation, especially with high positive end-expiratory end pressure (PEEP), is a well-known risk factor for pneumomediastinum and pneumopericardium. High intra-alveolar pressure causes susceptible alveoli to rupture allowing air to dissect along the bronchovascular sheaths towards the mediastinum [6]. Occasionally, pressure accumulation in the mediastinum causes air to escape into the pleural space (pneumothorax) or through weaknesses of the parietal pericardium (pneumopericardium). Air can also travel towards the thoracic inlet and into the neck soft tissue causing cervico-facial subcutaneous emphysema [6].

HFNC is a relatively newer form of ventilation used in the management of ARDS. HFNC generally delivers PEEPs lower than mechanical ventilation and some of this pressure escapes with mouth opening [11]. A few published cases described SPM in patients managed with HFNC, including that of a 77-year-old woman with influenza A and underlying chronic obstructive and interstitial lung disease after 4 days of HFNC at 60 L/min [11]. Similar incidents were noted in the pediatric population as well [12]. Both of our patients were on HFNC for several days prior to the development of SPM. It is unclear if HFNC causes barotrauma that contributed to the occurrence of SPM in our patients. Another therapy that has been used in the management of ARDS in COVID-19 patients is steroids [13]. Steroid’s use in the treatment of connective tissue diseases, such as dermatomyositis, has been postulated to contribute to the development of SPM by weakening the pulmonary interstitial tissue causing alveolar air leak [14]. Each of our patients received a cumulative dose of < 1 g methylprednisolone prior to the development of SPM. Although this is a small dose, further investigation is needed to determine if steroid use and cumulative dose play a role in the pathogenesis of SPM in COVID-19 patients.

Cough is a forceful expiration of air that causes sudden transient alveolar over-distention and occasionally rupture. It is a well-known risk factor for pneumomediastinum and pneumothorax [15]. Both of our patients had significant cough upon presentation to the hospital and during the early part of their hospital stay. The coughing spells, exerting strain upon the already damaged and weakened alveoli from COVID-19 pneumonia, could have directly contributed to the development of pneumomediastinum in our two patients.

Notably, both of our patients had history of underlying lung disease as well as obesity, which have been shown predispose patients to severe COVID-19 [16]. When COVID-19 outbreak data from Wuhan, China were examined, obese patients appeared to have higher severity of illness, and all-cause mortality [16]. Prior studies found obesity (BMI ≥30 kg/m^2^) to be an independent risk for pulmonary edema, DAD, and alveolar capillary hemosiderosis [17]. Taken together, one can postulate that obese COVID-19 patients, who are at risk for DAD, are also at higher risk for developing SPM.

In addition to identifying patient characteristics as potential risk factors for SPM, laboratory evaluation may have a predictive value. Lactate dehydrogenase (LDH) levels reflect the level of cell death due to plasma membrane damage. In the SARS outbreak, higher peak LDH was associated with SPM (mean 863 U/L) as compared with those without SPM (mean 583 U/L) [7]. Similarly, our two patients had high LDH peak prior to the onset of SPM (953 U/L and 1664 U/L for patient 1 and 2, respectively). If further study support this correlation, trending LDH levels may have a prognostic value in patients with COVID-19 pneumonia.

An interesting observation in our cases is the delayed appearance of SPM from onset of symptomatic infection (day 27 and day 18 for patient 1 and 2, respectively). SARS patients who developed SPM also showed similar delay, with a mean of 19.6 ± 4.6 days from symptom onset [7]. Therefore, clinicians should consider SPM in the differential diagnosis of new-onset pleuritic chest pain or unexpected respiratory deterioration after 2 weeks of illness in COVID-19 patients.

There are noteworthy diagnostic difficulties relating to SPM, especially in COVID-19 patients. Curvilinear radiolucency outlining the cardiac border is typical of pneumomediastinum on frontal CXR. This view, however, may only identify 50% of pneumomediastinum cases [18]. A lateral CXR, where gas is seen in the retrosternal space, is more sensitive [18]. When the diagnosis of pneumomediastinum remains uncertain, a chest CT is the modality of choice. Both lateral CXR and CT scans require transferring patients to and from the radiology suite. When applied to COVID-19 patients, these episodes of intra-hospital transport create potential breaches in infection control measures, a decision which must be weighed against the risk missing a diagnosis of SPM [19].

Finally, while SPM is usually self-limited in healthy individuals, patients with SARS and SPM had worse outcomes. Of those who developed SPM, 46% had simultaneous or new bilateral pneumathorax and most required bilateral chest tube insertion [7]. Ultimately, 38% progressed to intubation and 31% died [7]. Both of our COVID-19 patients eventually required intubation and mechanical ventilation and both suffered significant worsening of the subcutaneous emphysema on the ventilator and eventually expired. It is prudent to keep in mind that significantly elevated pressure in the mediastinal cavity (tension pneumomediastinum) and in the pericardium (tension pneumopericardium) can cause compression of intrathoracic structures leading to rapid respiratory and hemodynamic instabilities, complications that are more likely to occur with the use of positive pressure ventilation [19]. In such situations, a mediastinotomy with insertion of a drainage catheter should be strongly considered [20]. Although neither of our patients required immediate surgical intervention, the balance of maintaining adequate oxygenation while preventing exacerbation of the mediastinal emphysema while on mechanical ventilation proved to be challenging.

## Data Availability

Not applicable.

## Abbreviations

SPM: Spontaneous pneumomediastinum
SPP: Spontaneous pneumopericardium
ARDS: Acute respiratory distress syndrome
SARS: Severe Acute Respiratory Syndrome
CXR: Chest x-ray
CT: Computed tomography
HFNC: High flow nasal cannula
LDH: Lactate dehydrogenase
